# Heat health risk assessment analysing heatstroke patients in Fukuoka City, Japan

**DOI:** 10.1371/journal.pone.0253011

**Published:** 2021-06-21

**Authors:** Nishat Tasnim Toosty, Aya Hagishima, Ken-Ichi Tanaka

**Affiliations:** 1 Energy and Environmental Engineering, Interdisciplinary Graduate School of Engineering Sciences, Kyushu University, Kasuga-koen, Kasuga-shi, Fukuoka, Japan; 2 Department of Statistics, University of Dhaka, Dhaka, Bangladesh; 3 Faculty of Engineering Sciences, Kyushu University, Kasuga-koen, Kasuga-shi, Fukuoka, Japan; 4 Kyushu Environmental Evaluation Association, Fukuoka, Japan; Al Mansour University College-Baghdad-Iraq, IRAQ

## Abstract

**Background:**

Climate change, as a defining issue of the current time, is causing severe heat-related illness in the context of extremely hot weather conditions. In Japan, the remarkable temperature increase in summer caused by an urban heat island and climate change has become a threat to public health in recent years.

**Methods:**

This study aimed to determine the potential risk factors for heatstroke by analysing data extracted from the records of emergency transport to the hospital due to heatstroke in Fukuoka City, Japan. In this regard, a negative binomial regression model was used to account for overdispersion in the data. Age-structure analyses of heatstroke patients were also embodied to identify the sub-population of Fukuoka City with the highest susceptibility.

**Results:**

The daily maximum temperature and wet-bulb globe temperature (WBGT), along with differences in both the mean temperature and time-weighted temperature from those of the consecutive past days were detected as significant risk factors for heatstroke. Results indicated that there was a positive association between the resulting risk factors and the probability of heatstroke occurrence. The elderly of Fukuoka City aged 70 years or older were found to be the most vulnerable to heatstroke. Most of the aforementioned risk factors also encountered significant and positive associations with the risk of heatstroke occurrence for the group with highest susceptibility.

**Conclusion:**

These results can provide insights for health professionals and stakeholders in designing their strategies to reduce heatstroke patients and to secure the emergency transport systems in summer.

## 1. Nomenclature

As a number of symbols and notations have been used to describe the variables and the regression model, all of these symbols and notations are thus listed in Table 1.

**Table 1:** List of symbols and notations used in this work.

Symbol	Description
*T*_*mean*_	Daily mean temperature
*T*_*max*_	Daily maximum temperature
*T*_*min*_	Daily minimum temperature
*WBGT*_*mean*_	Daily mean WBGT
*WBGT*_*max*_	Daily maximum WBGT
*WBGT*_*min*_	Daily minimum WBGT
*T*_*rm*_	Running mean temperature
*α*	Weight parameter for calculating running mean temperature
*T*_od-k_	The average temperature of the *k*^*th*^ preceding day from the target day
*T*_rm1_	Running mean of the previous three days’ daily average temperatures
*T*_rm2_	Running mean of the previous four days’ daily average temperatures
*T*_w_	Time-weighted temperature of previous nine days
L	The number of days elapsed from the target day
S	Attenuation coefficient for calculating *T*_w_
*T*_*l*_	Daily mean temperature of the *l*^*th*^ day
*W*_*l*_	Weight used for *l*^*th*^ day
Δ*T*	Difference in the daily mean temperature between the target day and the previous day
Δ*T*_w_	Difference between the average temperature of the target day and time-weighted temperature
*N*	Total number of individuals (*i* = 1, 2, ⋯, *n*)
*P*	Total number of covariates (*j* = 1, 2, ⋯, *p*)
*μ*_*i*_	Mean response of *i*^*th*^ individual
x_i_	(*p* + 1) × 1 column vector of covariates for the *i*^*th*^ individual
	*x*_*i*_ = (1, *x*_*i*1_, *x*_*i*2_, ⋯, *x*_*ip*_)^*T*^
*β*	(*p* + 1) × 1 column vector of regression coefficients for the *i*^*th*^ individual
	*β* = (*β*_0_, *β*_1_, *β*_2_, ⋯, *β*_*p*_)^*T*^
*D*_w_	Working day
	$w=\left\{\begin{array}{ll} 1, & for\;working\;day\\ 0, & otherwise \end{array}\right.$

## 2. Introduction

Climate change is a perpetual global challenge [1] that requires prompt action. Although the long-term forecasts of global climate have suggested a steady increase in the global-scale annual temperature, worldwide experts have come to a consensus that the last three decades have been successively warmer than any preceding decades since 1850, as the Fifth Assessment Report (AR5) of the Intergovernmental Panel on Climate Change (IPCC) [2] reported. Furthermore, the urban heat island effects, which refer to the increase in temperature in the urban areas compared to the surrounding rural areas, manifests in various regions and cities accompanied by rapid urbanisation. Thus, urban people, being exposed to higher temperatures, suffer from the consequences of temperate weather [2, 3]. In light of this background, the health risks due to the temperature increase have been recognised as a serious future impact of climate change [4–6]. It should be noted that IPCC AR5 [7] identified the risk of death and illness from heat waves, especially in the vulnerable population of urban areas, as one of the eight major risks of global climate change. Therefore, the management of heat-related health issues has emerged as a defining challenge for the world as a whole, even including countries with a highly developed economy [8].

Japan has been recorded high temperatures since the 1990s and both the rate of temperature increase, and the duration of hot summer days in Japan have expanded from 1931 to 2016 [9]. Consequently, heatstroke, a malfunction of the central nervous system accompanied by a core body temperature of >41°C due to prolonged exposure to high temperature [10], has spurred as an inevitable health impact of frequent and intense hot weather in Japan [11]. The elevated number of heatstroke sufferers motivated Japanese researchers to determine the relationship of morbidity and mortality with temperature. The association between heatstroke-related emergency dispatches in Tokyo and daily maximum temperatures in July and August from 1993 to 1994 was analysed by Tamura et al. [12] (in Japanese). A statistical analysis of heatstroke-related ambulance dispatches from 2000 to 2009 in five major Japanese cities was also conducted, which revealed an increase in heat risk as a function of the daily maximum 3-hour apparent temperature [8]. The relationship between daily death counts from all causes in Fukuoka, Japan, and the daily mean air temperature based on 40-year data has been investigated [13]. In addition, the breakdown of heat stroke patients by sex and age has also been reported in previous studies highlighting the elevated risk of elderly people [14, 15].

Overall, past studies exploring various effect modifiers of the temperature-morbidity and mortality relationship indicated that the prominent modifier is age structure because of its proximate relationship with a person’s physiological response to temperature changes [16–19]. Since Japan is one of the countries that lead among aging societies, with 27% of its population over 65 years of age [20], various studies targeted heat-related health risks to elderly people. For instance, an elderly-focused survey conducted in eight urban areas of Japan identified a higher risk of indoor heatstroke and recommended the appropriate use of cooling appliances in houses as an intervention [11]. A case-control study was performed among people aged 65–84 years to examine the effectiveness of the heat health warning (HHW) broadcast accompanied by the delivery of water bottles labelled with HHW to houses as a preventive measure for heatstroke and the potential effectiveness of their proposed intervention was encountered in this study [21]. On the other hand, Kotani et al. [22] investigated the influence of temperature on ambulance dispatches due to not only heat stroke, but also all acute illnesses using the data from Fukuoka City, Japan, to determine the optimum temperature for various age groups [22].

Despite such age-specific studies in Japan, a comprehensive understanding of vulnerability to heatstroke among different age groups, as well as the influence of location and type of day (e.g., workday or holiday), have not been fully established. Further studies are needed to provide insight on health facility services for preventing heat-related illnesses. In fact, the heat waves of 2017 and 2018 in Japan led to an increase in heatstroke transporters to hospitals, which necessitated the development of national and regional strategies for establishing interventions and preventive measures against heatstroke [23]. In addition, a three-year project, the Regional Adaptation Consortium Project, was implemented in Japan in 2017 by the Ministry of the Environment, the Ministry of Agriculture, Forestry and Fisheries and the Ministry of Land, Infrastructure, Transport and Tourism. Through this project, local governments, universities, and research institutes jointly conducted surveys and analyses on the impact of climate change and potential adaptation measures, tailored to each region.

Under these circumstances, this study was originally planned as a part of this consortium project and aimed to contribute to establishing effective heat stroke prevention measures tailored to each local condition based on scientific data analysis. Furthermore, this study aimed to overcome the limitations of past studies conducted in Japan by identifying the determinants of HST to the hospitals in Fukuoka City, Japan, by adopting an age-structure decomposition. Fukuoka City was selected because it has the largest population among the cities in Kyushu Island, the southmost major island of Japan, and the local authority of this city has been working enthusiastically on developing measures against heat stroke. In addition, it was one of the six cities recorded with the highest wet-bulb globe temperature (WBGT) during the heatwaves of 2017 and 2018 [23].

The analysis procedure of this study was designed to facilitate detection of the susceptible group for not only Fukuoka City, but also for diversified geographic areas, to establish specific public health policies for reducing heat-related health problems, which is a global threat to human health. The increase in the proportion of elderly citizens worldwide has appeared to be an acute dilemma along with extreme weather conditions. According to the World Health Organization’s 2015 report [24], the proportion of the world population older than 60 years will double between 2015 and 2050. This has motivated research not only in Japan, but worldwide to identify the groups who are most vulnerable to heatstroke, accounting for varying effect modifiers. Studies aimed at identifying these vulnerable groups have been conducted in the Philippines [16, 25] and China [26], and have explored various effect modifiers of temperature-mortality associations. Most age-specific studies have determined a higher susceptibility of the elderlies to extreme temperatures owing to the deteriorating tendency of the thermoregulatory capacity of human body with respect to age advancement [27, 28]. Concurrently with age structure, several factors, such as the day of the week, holidays, activities of the patients, the places where patients faced heatstroke, etc. [19, 29–32] can also potentially affect the risk of heat-related illness, although there has been little comprehensive research encompassing all potential factors not only in Japan but also in other regions.

In this study, an attempt was made to determine potential risk factors of the heat stroke emergency transporters to the hospitals (HST) of Fukuoka, Japan, relying on the regression model appropriate to handle count data and explore the plausibility of previously detected risk factors. The data and variables are briefly discussed in the next section, along with a description of the regression model used in the study analyses. The third section presents an interpretation of the results from the overall analysis procedure. The fourth section includes a detailed discussion of the study findings and the final section presents the conclusions.

## 3. Materials and methods

### 3.1. Study site

The present study focused on heatstroke patients in Fukuoka City, which is located on the north coast of Kyushu, the southernmost of the major Japanese islands at a low latitude of 33°35ʹ and 130°24ʹ longitude. This city covers 343.39 km^2^ with more than half of the land owned in the built-up area, where the proportion of apartments (residential buildings including multiple dwelling units) is 78%. This highly populous city has a total population of 1,538,000, with a male to female ratio of 1:1.12, according to the national census of 2015 [33]. Elderly people, aged 65 and above, comprise 20.3% of the inhabitants of this city [33]. Among the employed residents, 85.2% work in a tertiary industry (e.g., teaching, nursing), which contributed to 90% of the gross domestic product of the city. Only 13.5% of employed residents of this city work outdoors in sectors, such as transportation, construction, machine operation, or agriculture. The weather in Fukuoka falls in a temperate and humid climatic region, accompanied with mild winters and hot, damp, and rainy summers. In Fukuoka City, the end of April is typically cooler, but transitions into the warm season. The warm season prevails until October, even though the summer season only spans from May to August. In fact, July and August are the warmest months in Fukuoka City with hot days, high humidity, and rainfalls.

### 3.2. Data

The data to be analysed in this study were obtained from the records of the patients who were sent to the hospitals of Fukuoka City due to heat-related illnesses and were originally provided by the Fukuoka City Fire Prevention Bureau. Prehospital emergency medical assistance and ambulance transportation services are provided by the Japanese local governmental fire defence headquarters. The ambulance service is free to all and can be accessed promptly by calling 119 [34]. The patients were administered prehospital treatment initially by trained ambulance crews, and their detailed information were recorded; such as their age, gender, type of accident, disease classification (sudden diseases are classified into ten classes according to the International Disease Classification ICD-10 established by WHO), degree of injury or illness (death, severe, moderate, minor injury, etc.) and the date and time of the dispatches. The ambulance staff also contacts the hospitals where the patients will be transported so that they can be diagnosed and provided with treatment immediately after the arrival [22]. This study included data on heatstroke-related ambulance dispatches, which were originally recorded annually by all seven fire departments in Fukuoka City between 1 April and 31 October from 2013 to 2018. The data were compiled as an anonymized dataset by the Fire Prevention Bureau, including observations on 3,124 heatstroke sufferers. All procedures performed in this study were in accordance with the 1964 Declaration of Helsinki and its later amendments and were approved by the Ethical Committee of Kyushu University. Obtaining written informed consent from patients was not required because all data provided by the Fukuoka City Fire Prevention Bureau were anonymised and this was a non-invasive observational study.

The daily statistics of the outdoor weather conditions for the targeted 1,284 days in total were available from other data sources, including the Japan Meteorological Agency. This time span was selected for the study with the aim of restricting the analyses to the warm season and suggesting specific intervention for this time period. Other data on the total population of Fukuoka City, arranged by the month of birth and classified according to five-year age structure and gender, were available from the 2015 National Census [33] conducted by the Ministry of Internal Affairs and Communications.

### 3.3. Variable selection

#### 3.3.1. Main covariates

**Fig 1:**
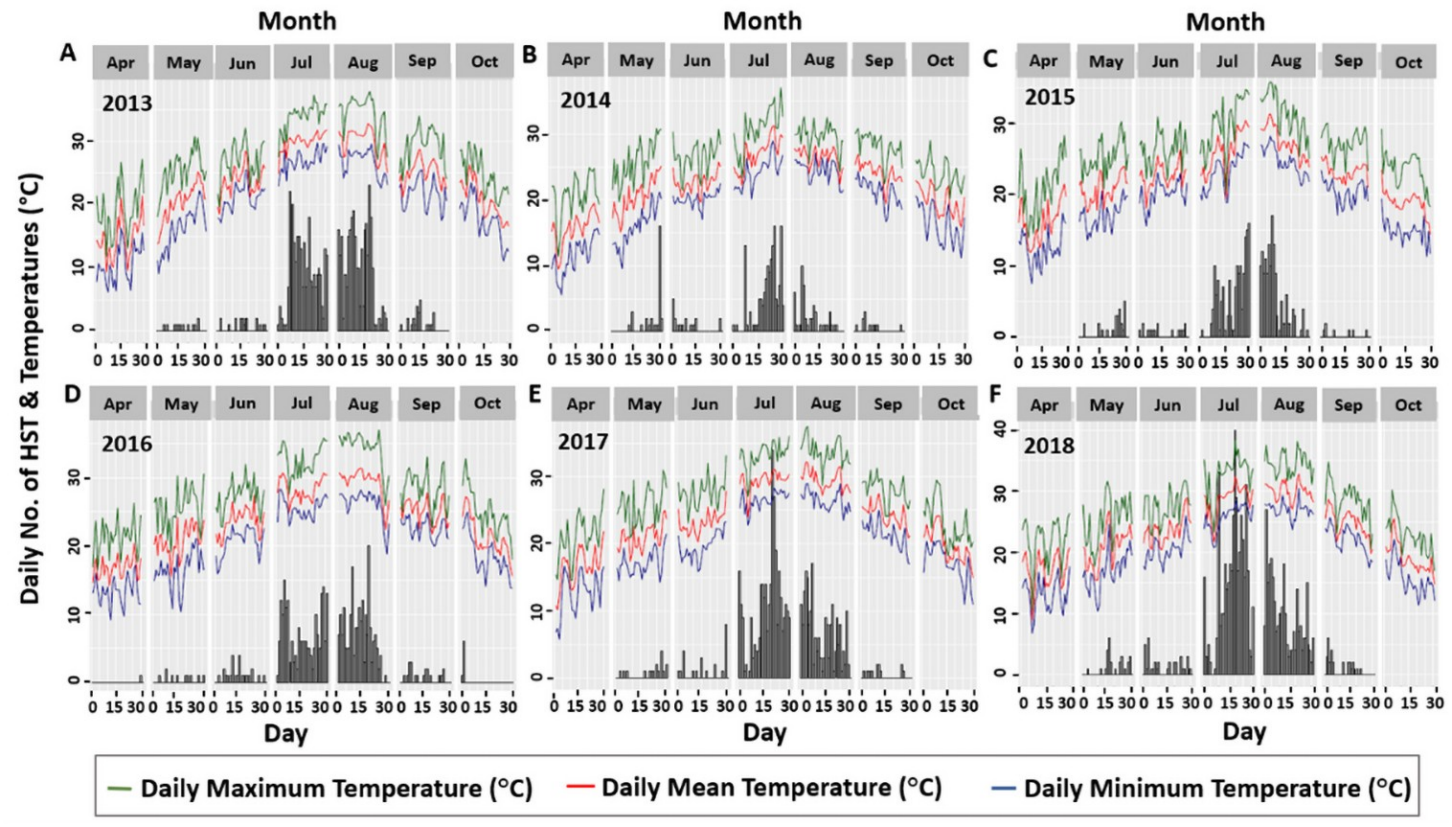
Association between daily temperature measures and number of HST from year 2013 to 2018.

Since most of the studies detected ambient temperature as one of the main causes of heatstroke occurrence [22, 26, 35, 36], the analysis procedure was initiated by visualising the relationship between the daily measures of temperature and the observed number of HST to hospitals. Three different measures: minimum, mean, and maximum hourly temperatures of a day were included in the analyses. Fig 1 displays the relationship between the daily temperatures and the number of HST.

**Fig 2:**
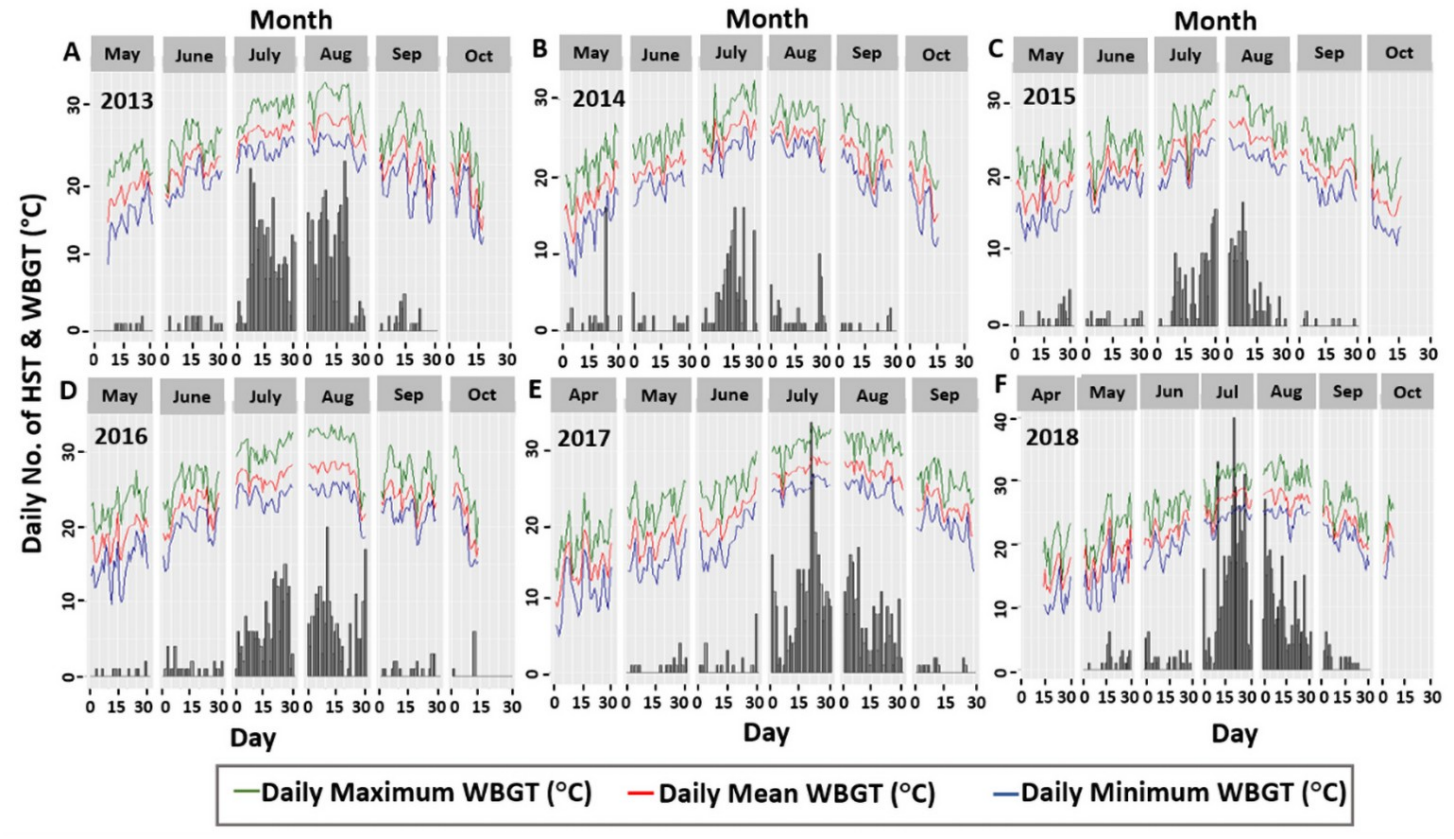
Association between daily WBGT estimates and number of HST from year 2013 to 2018.

This figure also depicts how this relationship evolved in the successive days of different months from the respective years. The number of HST per day is presented by the bars in Fig 1, while the daily temperatures have emerged from the line graphs. The positive association between the observed number of HST and daily measures of temperature is clearly shown in this figure, as the observed number of HST tended to vary with the daily temperatures. The researchers’ further attempt was to investigate the association between the daily number of HST and outdoor weather conditions in a broader way. To accomplish this, a new variable, termed wet-bulb globe temperature (WBGT), other than the daily temperature, was scrutinized. The new variable WBGT, which is a measure of heat stress in direct sunlight; considers temperature, humidity, wind speed, sun angle, and cloud cover in the calculation [37]. Thus, WBGT encompasses a variety of outdoor weather conditions. Fig 2 illustrates the association between the observed number of daily heatstroke carriers and daily maximum, mean, and minimum WBGT estimates, similar to the previous figure.

A strong positive correlation between the observed number of patients and the daily estimates of WBGT is depicted in Fig 2, which continued to prevail until 2018. It should also be noted that a sharp peak is observed in the bar chart of July for both 2017 and 2018, which is the health outcome of severe heat waves in 2017 and 2018 [23]. Thus, daily temperature and WBGT measures were selected as the main covariates for further analyses.

#### 3.3.2. Modified weather-related factors

Some modified weather-related factors were analysed with a view to projecting the effect of seasonality on the occurrence of heatstroke, focusing on human sensation and behaviour against temperature. Some of the adaptive thermal comfort studies analysed several variables to reflect the thermal comfort or adaptive behaviour of the human body. For example, Montazami et al. [38] analysed a variable named running mean outdoor temperature, which can appropriately capture people’s responses to the outdoor temperature. This temperature is formulated as:

$$T_{rm}=\left(1-\alpha \right)\left\{T_{od-1}+\alpha T_{od-2}+\alpha^{2}T_{od-3}+\cdots \right\},0<\alpha <1.$$ (1)

Here, *T*_*od*−*k*_ is the average temperature of the *k*^*th*^ preceding day from the target day. Thus, *T*_*rm*_ is the average temperature of a day, exponentially weighting the impact of the previous days’ temperatures. The impact of the temperature of the previous days tends to be less significant as time progresses, and this speed of decay depends on the value of *α*. ASHRAE (American Society of Heating, Refrigerating, and Air-Conditioning Engineers) [39] has recommended the value of *α* ranging from 0.6 to 0.9 and has suggested a fluctuation in the proper value of *α* according to climate zones. Ono et al. [40] (written in Japanese) considered seasonality to analyse the occupant behaviour of air-conditioner use with respect to outdoor thermal conditions. In this study, the authors concluded that the air-conditioner usage time can be more accurately predicted by the weighted average temperature incorporating the history of the outside temperature for the past several days to about a week, rather than the average temperature of the current day. The time-weighted outdoor temperature was determined as follows:

Tw=∑l=09TlWl∑l=09Wl,Wl=exp-ls. (2)

In this equation, *s* represents the relative strength of the memory. The value of *s* was determined to be 3.27 by Ono et al. [40].

To incorporate seasonality in the study’s analysis procedure, the researchers included some generalised statistics related to climatic conditions as the explanatory variable along with daily temperatures or WBGT estimates. The modified variables that were incorporated are as follows:

- Running mean of the previous three days’ daily average temperatures (with *α* = 0.8, according to Montazami et al. [38])
- Running mean of the previous four days’ daily average temperatures (with *α* = 0.8, according to Montazami et al. [38])
- Time-weighted temperature (according to Ono et al. [40])
- Difference in the daily mean temperature between the target day and the previous day
- Difference between the average temperature of the target day and time-weighted temperature

### 3.4. Modelling approach

Due to the association illustrated in Figs 1 and 2, an appropriate regression model for count data was developed, including the daily measures of temperature and WBGT. The Poisson regression model is a common choice for researchers to analyse count data [41–43]. However, the equidispersion assumption, which represents the equal population mean and variance, restricted the Poisson regression model. Alternatively, real-life data rarely exhibit a highly equal mean-variance relationship. In most cases, the variance exceeds the mean, which is the situation when overdispersion occurs. The Poisson regression model is inappropriate for handling such over-dispersed data, and the negative binomial (NB) regression model performs proper rigour with the estimation procedure in the presence of overdispersion [44–46]. This model has been extensively used by researchers [47–49] to manage over-dispersed count data attributable to rare events, such as different types of infectious and non-infectious diseases. The NB regression model, a generalisation of the Poisson regression model, incorporates overdispersion in the estimation procedure through the inclusion of an additional parameter, which is termed the overdispersion parameter [50, 51]. In the NB regression model, the mean of the outcome variable is determined by a set of covariates, and this relationship can be expressed by using the following functional form:

$$ln\mu_{i}=x_{i}^{T}\beta ;\;i=1,2,\cdots ,n.$$

**Fig 3:**
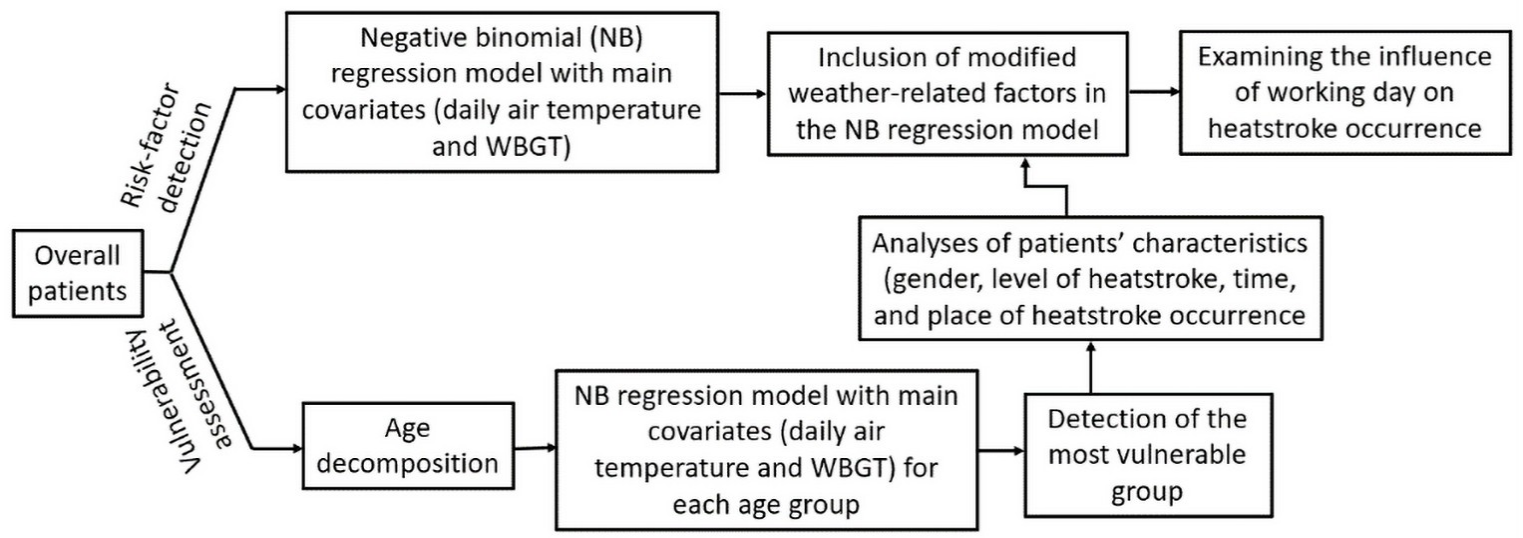
Flowchart of the research methodology.

Fig 3 shows the flowchart for all the analyses conducted.

First, the HST for all ages were analysed using NB regression models including the main covariates, daily temperatures, and WBGT, with the goal of detecting risk factors for heatstroke. In addition, the modified weather-related factors were incorporated in the NB regression models with main covariates. Subsequently, an analysis was conducted on age structure decomposition based on the observed daily number of varying aged HST per 1000 residents of Fukuoka, Japan. An NB regression model was used to determine the group who is most vulnerable to heatstroke by including the main covariates for each specified age group and predicting mean number of daily HST. The most susceptible group was then analysed to identify characteristics, such as gender, level of heatstroke, and time and place of heatstroke occurrence. NB regression models with both main covariates and modified weather-related factors were also used to detect potential factors of heatstroke risk for the most susceptible group. Finally, the influence of the type of day, whether it is a working day or not, on heatstroke occurrence was investigated for all patients and the most vulnerable group.

## 4. Results

Before conducting any regression model, the researchers determined the mean and variance of the daily HST counts for each year, which are presented in Table 2.

**Table 2:** The descriptive statistics of the daily number of HST in Fukuoka from 2013 to 2018.

Year	Mean daily HST counts	Variance of daily HST counts
2013	2.96	28.18
2014	1.21	8.08
2015	1.65	11.62
2016	2.23	14.11
2017	2.71	25.90
2018	3.84	49.55
Overall	2.43	23.56

Table 2 clearly demonstrates that the variance exceeded the mean number of daily HST from 2013 to 2018, which was also true for the overall statistics. Hence, the NB regression model was selected for the analysis of the daily HST counts.

### 4.1. Exploratory analyses

The descriptive statistics of all the selected covariates, both main and modified weather-related factors, are presented in Table 3. The approximate correlation of these covariates with the response of interest (daily number of HST) along with the p-values for the statistical significance of these correlations, are incorporated in this table.

**Table 3:** Descriptive statistics and approximate correlations with response variable accompanied by the p-values.

Covariates	Mean	Standard Deviation	Range	Correlation
				(p-value)
***T***_***max***_	27.29	5.02	(10.6, 38.3)	0.651***
***T***_***mean***_	23.09	4.83	(8.9, 32.8)	0.643***
***T***_***min***_	19.83	5.20	(5.6, 30.5)	0.600***
***WBGT***_***max***_	26.12	4.18	(12.5, 34.2)	0.645***
***WBGT***_***mean***_	22.50	4.12	(8.9, 26.9)	0.624***
***WBGT***_***min***_	19.77	4.54	(4.9, 26.9)	0.559***
***T***_**rm1**_	11.32	2.28	(4.9, 15.9)	0.602***
***T***_**rm2**_	13.71	2.72	(6.25, 19.12)	0.601***
***T***_**w**_	23.42	4.38	(11.7, 32.0)	0.629***
**Δ*T***	0.006	1.59	(-7.2, 5.2)	0.109***
**Δ*T***_**w**_	0.03	1.29	(-4.9, 4.9)	0.194***

***: p-value<0.001,

**: p-value<0.01,

*: p-value<0.05,

ʹ: p-value<0.1.

The above table depicts the statistical significance of the association between the response and all the selected covariates, which indicates that the covariates are suitable to be included in the model of interest if there is no evidence of multicollinearity among these covariates.

### 4.2. NB regression models

Initially, four different NB regression models were conducted with four different independent variables. Daily minimum temperature and WBGT estimates were excluded from these models because heatstroke is not usually expected to occur at the minimum temperature of a day, as well as in the case of daily minimum WBGT estimates. Table 4 indicates the results of these regression models, including the incidence rate ratio (IRR), which is the exponent of the estimated coefficients of interest.

**Table 4:** Results of four different NB regression models with main covariates.

Models		Model I	Model II	Model III	Model IV
Covariates		*T*_*mean*_	*T*_*max*_	*WBGT*_*mean*_	*WBGT*_*max*_
**Estimates of IRR**	$e^{\hat{\beta_{0}}}$	2.12×10^−5^***	5.83×10^−6^***	5.83×10^−6^***	1.28×10^−5^***
	$e^{\hat{\beta_{1}}}$	1.543***	1.514***	1.550***	1.534***
**AIC**		3233	3213	3317.7	3239.4
**RMSE**		0.9008	0.8707	1.0821	0.9786
**MAE**		0.7161	0.7062	0.8302	0.7977
**MSE**		0.8115	0.7581	1.0569	0.9577
**Sample Size**		1284	1284	1032	1032

***: p-value<0.001,

**: p-value<0.01,

*: p-value<0.05,

ʹ: p-value<0.1.

All the results using the NB regression model were obtained using the R package MASS, which relied on the maximum likelihood estimation approach. In each of these four models, the covariate of interest was determined to have a positive and highly significant association with the log of the mean response. Four different model selection criteria were used in this study: Akaike’s information criteria (AIC), root mean square error (RMSE), mean absolute error (MAE) and mean square error (MSE). These four models cannot be compared solely based on the lowest values of these criteria, as some of these criteria (RMSE, MAE, and MSE) tend to increase with a decrease in sample size. The models conducted with samples of the same size can only be compared by relying on these four criteria. Hence, the NB regression models with covariate daily maximum temperature and WBGT (Models II and IV) were found to provide a better fit for the data. The IRR was used for convenient interpretation of the effect estimates. The IRR of the daily maximum temperature is 1.514, which is defined as: For every 1°C increase in the highest temperature of a day, the mean number of daily HST also increases by (1.514 − 1) × 100% = 51.4%. All other models can be interpreted in a similar manner.

Before incorporating the modified variables into the models with the main explanatory variables, it is necessary to determine the correlation between these covariates. The rationale is to determine the multicollinearity problem, which may arise if the covariates of a model have a high linear correlation among themselves because it may mislead the model conclusions. The correlation coefficients and p-values are presented in Table 5.

**Table 5:** Correlations between the main covariates and modified weather-related factors.

Covariates	*T*_rm1_	*T*_rm2_	*T*_w_	Δ*T*	Δ*T*_w_
***T***_***mean***_	0.928***	0.923***	0.959***	0.159***	0.282***
***T***_***max***_	0.851***	0.847***	0.889***	0.272***	0.379***
***WBGT***_***mean***_	0.901***	0.897***	0.936***	0.102**	0.239***
***WBGT***_***max***_	0.841***	0.839***	0.887***	0.209***	0.321***

***: p-value<0.001,

**: p-value<0.01,

*: p-value<0.05,

ʹ: p-value<0.1.

All modified variables were found to be significantly correlated with the set of explanatory variables. Although the covariates Δ*T* and Δ*T*_*W*_ were significantly correlated with the daily temperature and WBGT measures, the correlation coefficients in Table 5 were weak. Therefore, these two modified variables were added to the NB regression model with each of the main explanatory variables to capture the influence of seasonality on the daily HST counts.

Table 6 presents the results of the eight NB regression models.

**Table 6:** Results of eight different NB regression models with main covariates and modified weather-related factors.

Models		V	VI	VII	VIII	IX	X	XI	XII
Covariates		*T*_*mean*_	*T*_*max*_	*WBGT*_*mean*_	*WBGT*_*max*_	*T*_*mean*_	*T*_*max*_	*WBGT*_*mean*_	*WBGT*_*max*_
		Δ*T*	Δ*T*	Δ*T*	Δ*T*	Δ*T*_w_	Δ*T*_w_	Δ*T*_w_	Δ*T*_w_
**Estimates of IRR**	$e^{\hat{\beta_{0}}}$	2.11×10^−5^***	5.83×10^−6^***	4.53×10^−5^***	1.33×10^−5^***	2.44×10^−5^***	6.27×10^−6^***	5.52×10^−5^***	1.49×10^−5^***
	$e^{\hat{\beta_{1}}}$	1.542***	1.514***	1.550***	1.531***	1.533***	1.511***	1.536***	1.522***
	$e^{\hat{\beta_{2}}}$	1.117***	0.986	1.181***	1.084**	1.095**	0.999	1.239***	1.184***
**AIC**		3214.2	3214.3	3275.5	3231.8	3222.2	3210.2	3265.3	3210.2
**RMSE**		0.8950	0.8727	1.0162	0.9757	0.9141	0.8880	1.0916	0.9793
**MAE**		0.7105	0.7087	0.8188	0.7935	0.7358	0.7278	0.825	0.7969
**MSE**		0.8010	0.7616	1.0327	0.9519	0.8356	0.7885	1.0395	0.9591
**Sample Size**		1278	1278	1031	1031	1230	1230	1023	1023

***: p-value<0.001,

**: p-value<0.01,

*: p-value<0.05,

ʹ: p-value<0.1.

If a comparison among the eight different NB regression models (Table 6) is performed based on the model selection criteria considering the equal sample size, the models with maximum temperatures and WBGT estimates (Models VI, VIII, X and XII) are found to provide a better fit for each pair. However, Models VI and X indicate the insignificant association between the modified variables (Δ*T* and Δ*T*_*W*_) and the daily number of HST. This means that these two models are no longer different from the previous NB model with daily maximum temperature (Model II). All the models in Table 6, except Models VI and X, depict the positive and statistically significant associations between the corresponding covariates and the response of interest. The IRRs can be interpreted in a manner which is similar to that mentioned earlier. For example, the IRR from Model V indicates that a 1°C increase in the daily mean temperature results in an increase in the average number of HST by (1.542 − 1) × 100% = 54.2% on a day if the difference between the daily mean temperature of that specific day and the day before is maintained at a constant level. The daily mean HST counts increases by (1.117 − 1) × 100% = 11.7% for a 1°C increase in the difference of the daily mean temperature between the target day and the previous day, while the average temperature of the target day is constant.

### 4.3. Breakdown of HST

**Fig 4:**
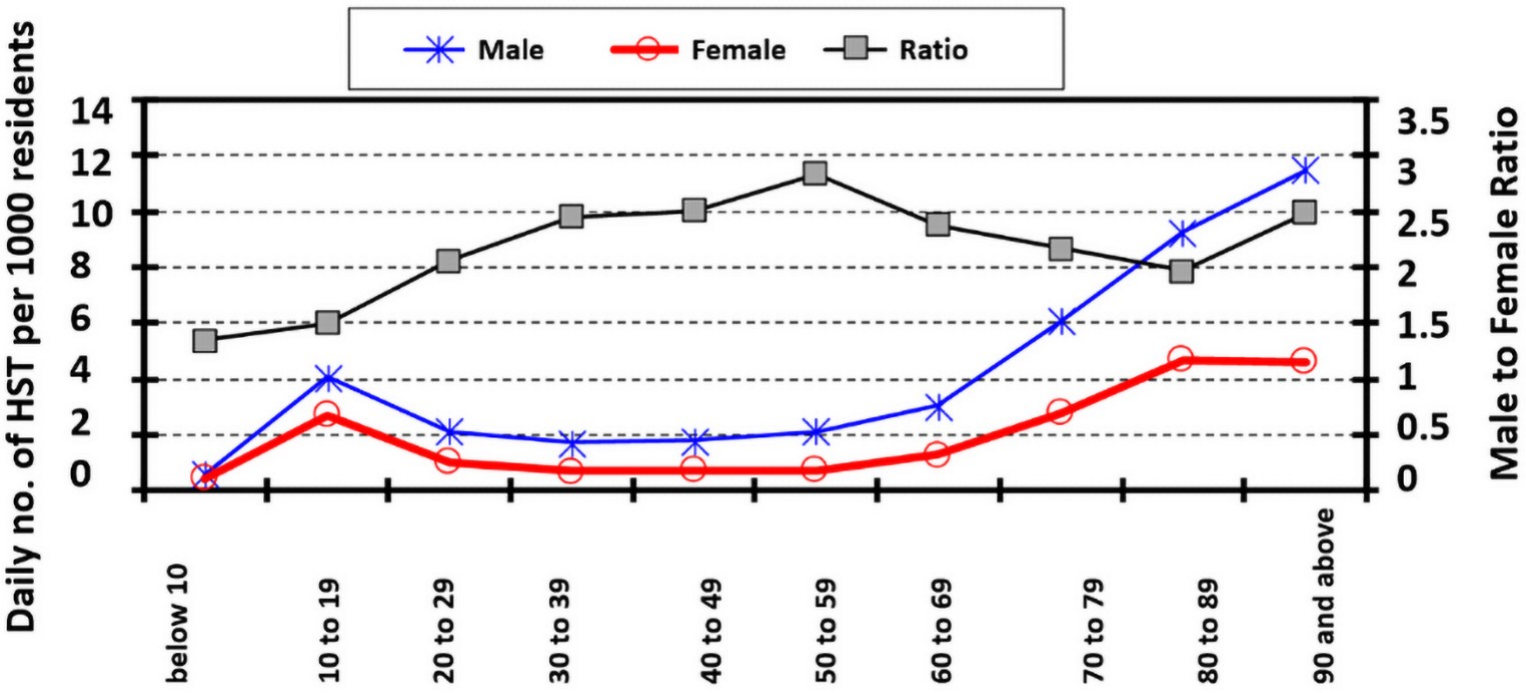
Decomposition of the daily HST counts per 1,000 residents of Fukuoka City by age and gender.

Since one of the main objectives of this study was to scrutinise the age structure of heatstroke patients with a view to pinpointing the sub-population with the highest degree of susceptibility, the breakdown of the patients was a prerequisite. Fig 4 depicts the breakdown of the daily number of HST per 1,000 residents of Fukuoka City with respect to a 10-year age structure accompanied by gender. The male-to-female patient ratio is also illustrated in Fig 4.

It can be observed from the figure that the number of male HST per 1,000 residents was higher than that of the female HST for all age groups except for the group less than 10 years old. Both male and female patient numbers increased steeply from the age of 60 years. The number of male patients continued to increase with a greater slope even after the age of 90 years, while a stable position was achieved by the number of female patients in that period. Due to such an illustration of the breakdown of HST counts, further analysis was conducted in four age groups that were not mutually exclusive. The first focused on younger patients and consisted of patients aged < 60 years. In this group, all the groups of young patients were accumulated as no noticeable pattern of change was observed in the groups of patients. Among the elderly patients, three groups were considered to identify the most fragile people, and these groups were patients aged ≥60 years, ≥65 years and ≥70 years. The analysis procedure was then followed by conducting separate models with explanatory variables, daily average and maximum temperatures and WBGT estimates. The results obtained from the NB regression models for the four age groups, with daily temperatures and WBGT estimates are shown in Tables 7 and 8, respectively.

**Table 7:** Results of NB regression model for different age groups with daily mean and maximum temperatures.

Age Group		<60 Years		≥60 Years		≥65 Years		≥70 Years	
Covariates		*T*_*mean*_	*T*_*max*_	*T*_*mean*_	*T*_*max*_	*T*_*mean*_	*T*_*max*_	*T*_*mean*_	*T*_*max*_
**Estimates of IRR**	$e^{\hat{\beta_{0}}}$	0.010***	0.005***	0.004***	0.004***	0.007***	0.007***	0.013***	0.013***
	$e^{\hat{\beta_{1}}}$	1.231***	1.221***	1.262***	1.224***	1.236***	1.204***	1.207***	1.178***
**AIC**		1817.4	1802.9	1667.9	1694.6	1535.1	1559.5	1354.8	1370.1
**RMSE**		0.936	0.920	0.900	0.902	0.896	0.900	0.880	0.885
**MAE**		0.750	0.734	0.697	0.695	0.695	0.694	0.671	0.673
**MSE**		0.876	0.847	0.810	0.813	0.802	0.809	0.775	0.783
**Sample Size**		430	430	427	427	405	405	374	374

***: p-value<0.001,

**: p-value<0.01,

*: p-value<0.05,

ʹ: p-value<0.1.

**Table 8:** Results of NB regression model for different age groups with daily mean and maximum WBGT estimates.

Age Group		<60 Years		≥60 Years		≥65 Years		≥70 Years	
Covariates		*WBGT*_*mean*_	*WBGT*_*max*_	*WBGT*_*mean*_	*WBGT*_*max*_	*WBGT*_*mean*_	*WBGT*_*max*_	*WBGT*_*mean*_	*WBGT*_*max*_
**Estimates of IRR**	$e^{\hat{\beta_{0}}}$	0.017***	0.007***	0.004***	0.004***	0.006***	0.006***	0.012***	0.011***
	$e^{\hat{\beta_{1}}}$	1.230***	1.231***	1.292***	1.254***	1.265***	1.230***	1.230***	1.202***
**AIC**		1856.6	1843.2	1687	1709.8	1549.6	1569.5	1364.1	1377.2
**RMSE**		0.960	0.945	0.922	0.916	0.914	0.910	0.894	0.893
**MAE**		0.777	0.763	0.743	0.722	0.727	0.707	0.694	0.680
**MSE**		0.922	0.892	0.850	0.839	0.835	0.828	0.799	0.797
**Sample Size**		427	427	427	427	405	405	374	374

***: p-value<0.001,

**: p-value<0.01,

*: p-value<0.05,

ʹ: p-value<0.1.

All these models from Tables 7 and 8 depict positive and highly significant associations between the daily number of HST and the daily measures of temperature and WBGT. These models can also be interpreted by using the method discussed above.

**Fig 5:**
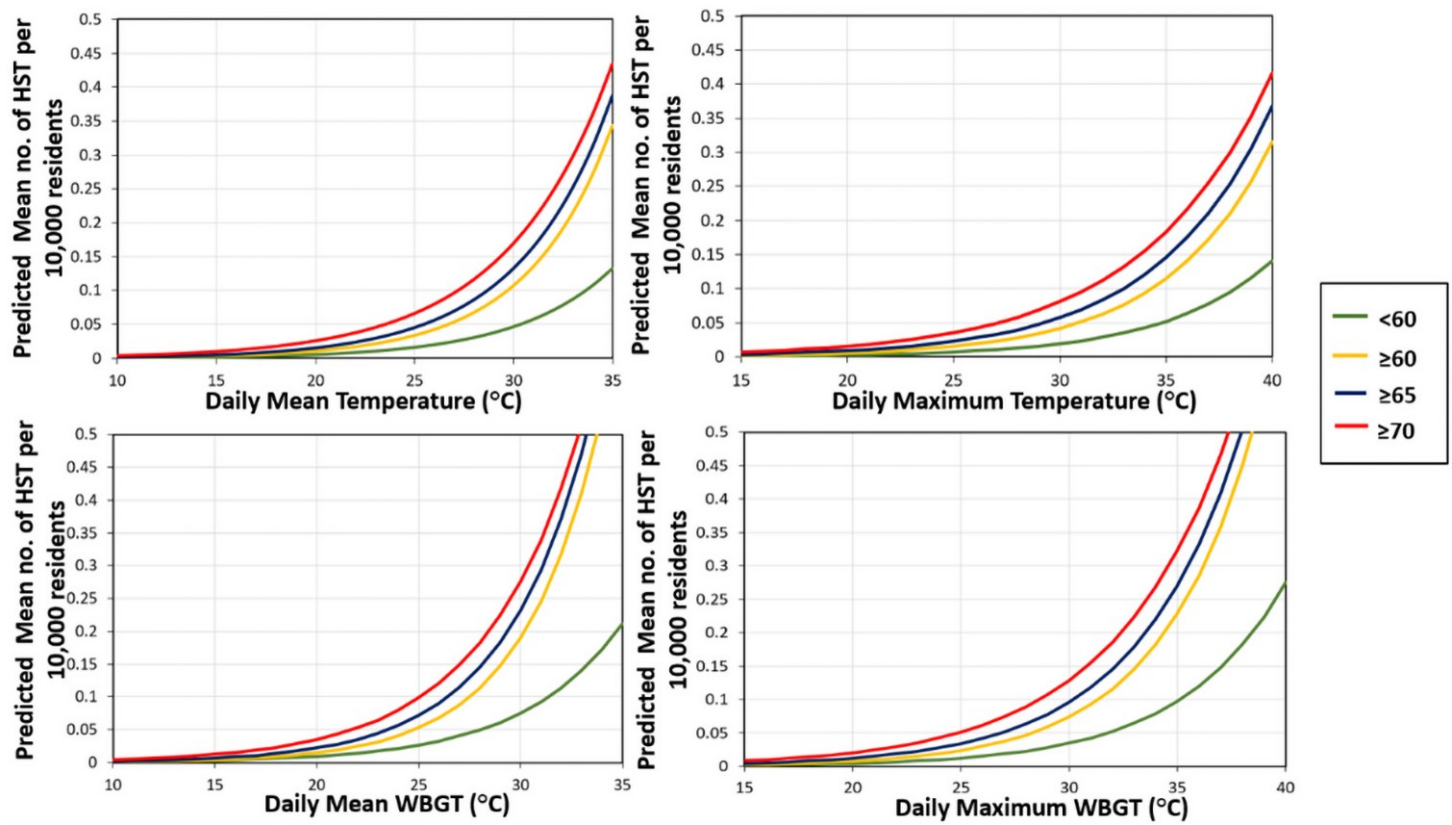
Comparison between the predicted mean number of HST per 10,000 residents of Fukuoka City among different age groups.

The comparison among the number of daily HST, predicted for the daily measures of temperature and WBGT among different age groups, may serve as a valid assessment tool in determining the highest susceptible group. Such a comparison is demonstrated in Fig 5, which shows the predicted mean number of daily HST per 10,000 residents of Fukuoka City using the results of the models presented in Tables 7 and 8.

Fig 5 shows that as the age group of the patients increased, the predicted curves moved towards higher positions, and the same pattern was observed in all the graphs of this figure. Such a pattern in the graphs clearly indicates that the people of Fukuoka City aged 70 years or above are most vulnerable to heatstroke.

#### 3.3.1. Characteristics analysis of vulnerable group

**Fig 6:**
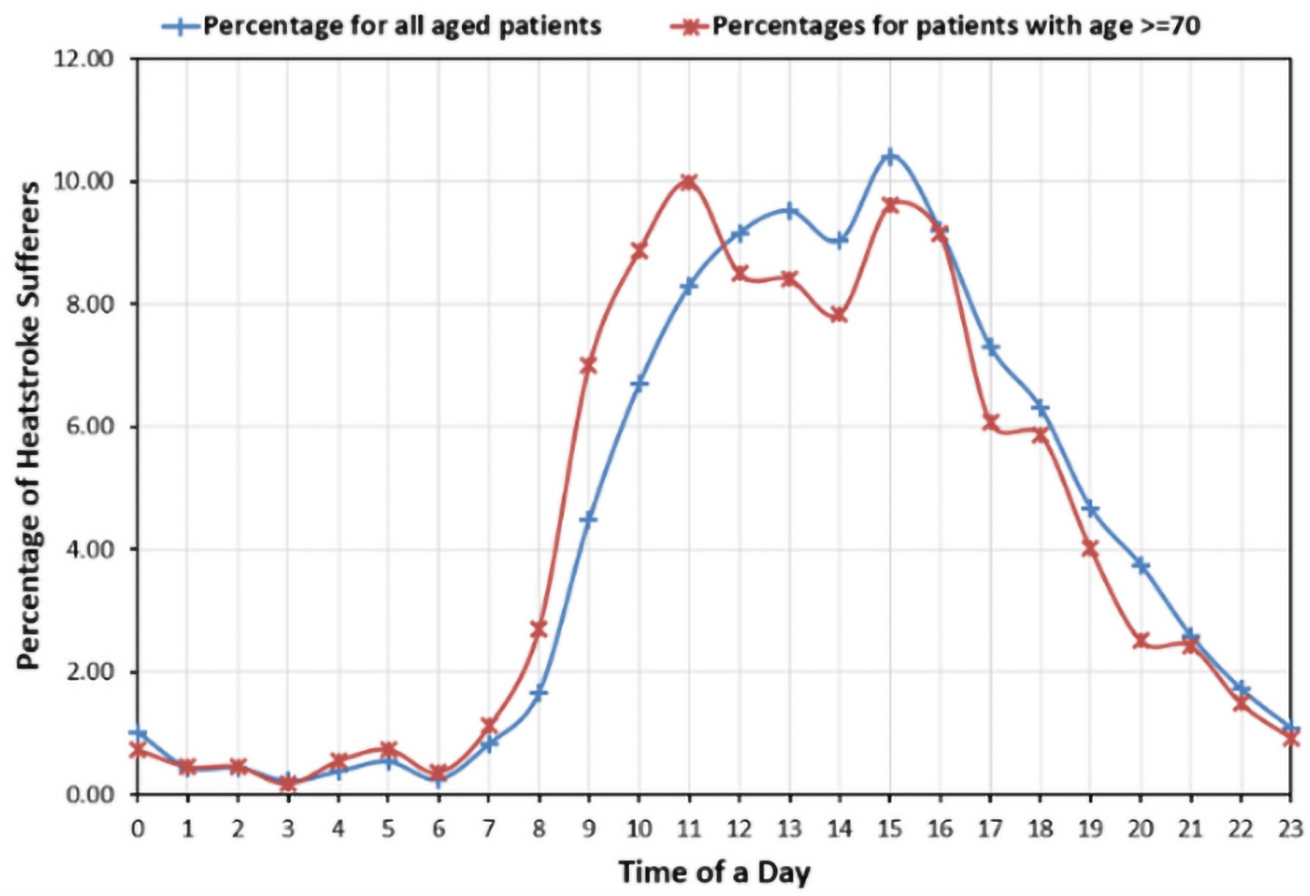
Percentage distribution for the time of heatstroke occurrence for overall patients and vulnerable group.

Various characteristics, such as the time and place of heatstroke occurrence, gender, and the level of heatstroke of the most vulnerable people were analysed using bar charts. Fig 6 illustrates the time of heatstroke occurrence for both, overall patients, and those of the most vulnerable group.

Fig 6 depicts the lowest percentage of heatstroke occurrence during midnight for patients of all ages including those aged 70 or more. The morning time period is encountered to be riskier for the elderlies than the overall patients, whereas afternoons and evenings appeared to have a higher percentage of heatstroke occurrence for overall patients than for the vulnerable group. However, both these groups experienced heat-related illness more frequently during the daytime, specifically from 10 a.m. to 4 p.m.

**Fig 7:**
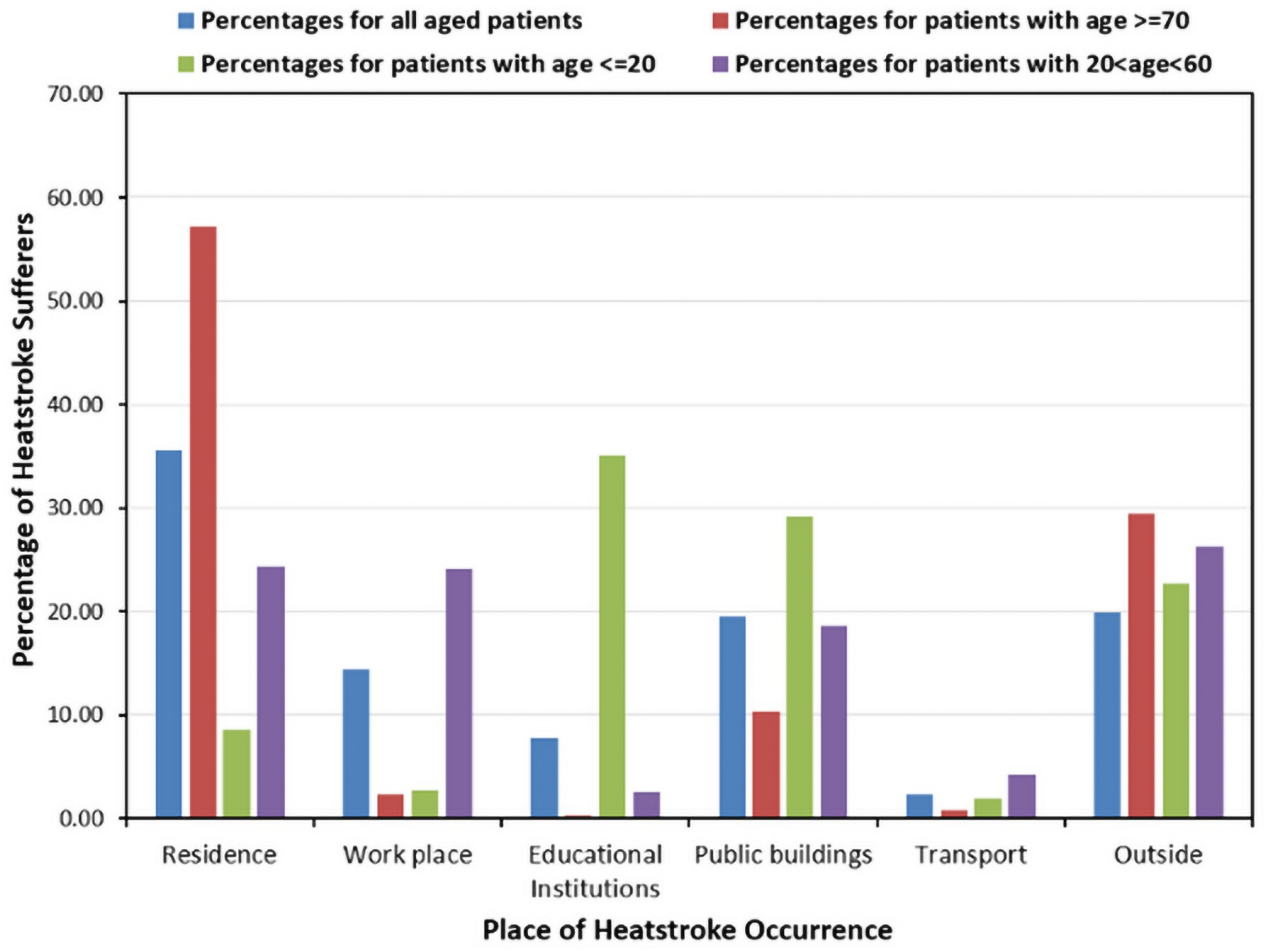
Percentage distribution for the place of heatstroke occurrence for the varying aged patients.

The locations of heatstroke occurrences were analysed by comparing the percentages among the four groups: patients of all ages, patients among students (aged ≤20), patients among working people (20<age<60) and those among the most vulnerable group (aged ≥70 years). This comparison is illustrated in Fig 7.

The above figure shows the highest percentage of heatstroke occurrence for all patients in their residences. The place with the second highest position for these patients was outside and public buildings. Any places outside a building or educational institution, such as a street, mountain, park, stadium, gallery, field, river, workplaces outside office or plants, etc. were accumulated in the category “outside,” while a public building referred to any building open for the masses. Most of the patients from the vulnerable group experienced heat-related illnesses in their residences, which was followed by outside places. Educational institutions encountered the highest number of young heatstroke sufferers aged 20 years or less. The young generation was also prone to heatstroke in public buildings and outside places with high percentages. For the working people aged between 20 and 60 years, the riskiest place was outside areas, while residences and workplaces secured the second highest position with respect to the risk of heatstroke. Moreover, Fig 7 identifies hazardous places for heatstroke, portraying considerable variation in the health hazards of these places among the different age groups.

**Fig 8:**
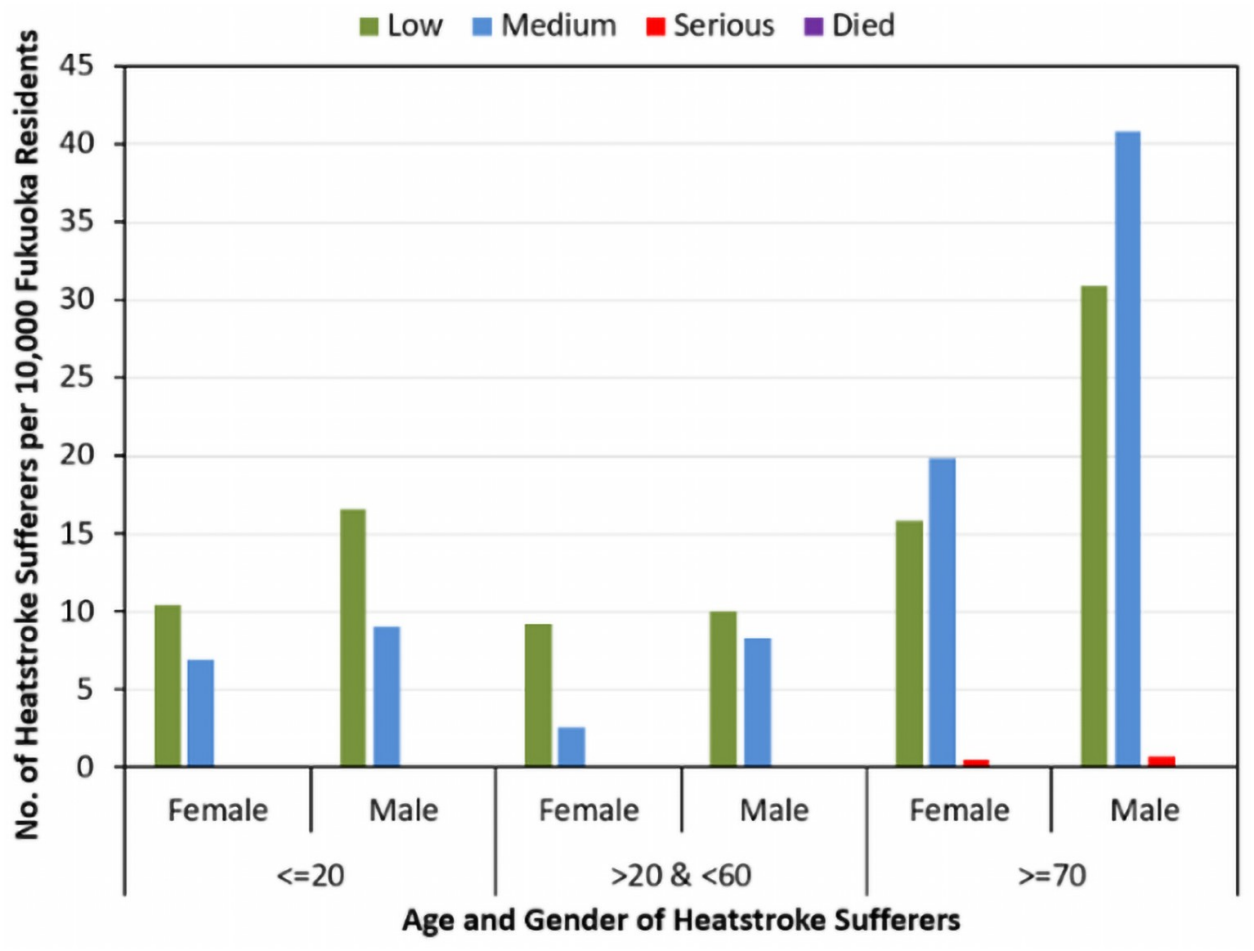
Percentage distribution for varying level of heatstroke occurrence among patients of different age and gender.

Fig 8 presents a comparison of the percentages of heatstroke occurrence with varying levels among heatstroke patients of different ages and genders.

This figure clearly shows that most of the heatstroke cases in these studies were confined to low and medium levels. Only a few people from the most vulnerable group encountered a serious level of heatstroke. The category “died” is missing in this figure, as only one female heatstroke patient in this study died, which comprised a rate too small to be evident in the figure. A comprehensive comparison among the categories of Fig 8 denotes the dominance of male patients over the number of females at all levels of heatstroke, indicating a higher susceptibility of male residents of Fukuoka City, Japan. Patients from both younger and working groups of either gender experienced a low level of heatstroke with a higher number compared to that of the medium level, however the number of working males presenting with heatstroke at the medium level was much higher than that of their female counterparts. The vulnerable group had the highest number of heatstroke sufferers among all other age groups, which was accompanied by a greater number of medium-level heatstroke sufferers than any other level. This suggests that not only the risk of heatstroke but also its severity increases with age.

#### 3.3.2. Potential factors determination for vulnerable group

In order to detect potential determinants of heatstroke occurrence among the most vulnerable people, the modified factors, **Δ*T*** and **Δ*T***_***W***_, were included in the models with main covariates for people aged 70 years and above. Table 9 lists the results of the models.

**Table 9:** Results of NB regression models with main covariates and modified weather-related factors for patients aged ≥70 years.

Covariates		*T*_*mean*_	*T*_*max*_	*WBGT*_*mean*_	*WBGT*_*max*_	*T*_*mean*_	*T*_*max*_	*WBGT*_*mean*_	*WBGT*_*max*_
		Δ*T*	Δ*T*	Δ*T*	Δ*T*	Δ*T*_*w*_	Δ*T*_*w*_	Δ*T*_*w*_	Δ*T*_*w*_
**Estimates of IRR**	$e^{\hat{\beta_{0}}}$	0.013***	0.012***	0.011***	0.011***	0.013***	0.012***	0.011***	0.011***
	$e^{\hat{\beta_{1}}}$	1.207***	1.179***	1.232***	1.202***	1.207***	1.179***	1.231***	1.202***
	$e^{\hat{\beta_{2}}}$	1.002	0.961	1.026	0.996	1.003	0.983	1.067*	1.054
**AIC**		1356.8	1370.2	1365.3	1379.1	1356.8	1371.8	1362.3	1376.7
**RMSE**		0.880	0.883	0.893	0.893	0.880	0.884	0.892	0.893
**MAE**		0.671	0.669	0.694	0.680	0.671	0.671	0.695	0.680
**MSE**		0.775	0.780	0.798	0.797	0.775	0.782	0.796	0.797
**Sample Size**		374	374	374	374	374	374	374	374

***: p-value<0.001,

**: p-value<0.01,

*: p-value<0.05,

ʹ: p-value<0.1.

Among the second set of variables, essentially, the modified variables, only Δ*T*_*w*_ was found to be significant in only one model, which was the model with mean WBGT and Δ*T*_*w*_. The estimated IRRs of this model indicate that for every 1°C increase in the daily average WBGT, the daily mean HST count increases by 23.1% if the difference between the mean temperature of that specific day and previous nine days’ time-weighted temperature is fixed. Similarly, for a constant daily mean WBGT, the average number of daily HST increases 6.7% owing to a 1°C increase in the difference between the current day’s mean temperature and the time-weighted temperature of the previous nine days. This implies that not only the current day’s weather conditions but also the average temperature of the previous days, up to the past nine consecutive days, can have a positive impact on the heatstroke occurrence for those who are aged 70 years and more people in Fukuoka City.

### 4.4. Influence of working day

Thereafter, an attempt was made to project the influence of a working day, in other words, whether the day of interest was a working day or not, on heatstroke occurrence. On that account, the working day was included as a binary variable, which took a value of 1 for working days and 0 otherwise, in the models of interest. However, no significant difference in the predicted mean number of HST was detected depending on whether or not the day was a working day from either the models conducted for all aged HST or the models for the three elderly age groups (≥60, ≥65, and ≥70). The models used for the younger HST (<60) resulted in a statistically significant and negative association between the daily HST count and a working day. Table 10 presents the results obtained from the models with working days concentrated on heatstroke patients aged below 60 years, which provided the best fit.

**Table 10:** Results of the best models encountered with significant effect of working day.

Age Group		<60 Years		≤20 Years	
Covariates		*T*_*max*_	*WBGT*_*max*_	*T*_*max*_	*WBGT*_*max*_
		*D*_*w*_	Δ*T*_*w*_	Δ*T*_*w*_	Δ*T*_*w*_
			*D*_*w*_	*D*_*w*_	*D*_*w*_
**Estimates of IRR**	$e^{\hat{\beta_{0}}}$	0.005***	0.007***	0.099***	0.148***
	$e^{\hat{\beta_{1}}}$	1.222***	1.234***	1.100***	1.094***
	$e^{\hat{\beta_{2}}}$	0.865*	1.142***	1.074ʹ	1.114*
	$e^{\hat{\beta_{3}}}$		0.875ʹ	0.805*	0.811*
**AIC**		1800.5	1826.9	949.57	953.38
**RMSE**		0.920	0.943	0.858	0.872
**MAE**		0.727	0.762	0.548	0.663
**MSE**		0.8455	0.889	0.737	0.760
**Sample Size**		430	427	288	285

***: p-value<0.001,

**: p-value<0.01,

*: p-value<0.05,

ʹ: p-value<0.1.

The performance of the models was assessed based on the included model selection criteria, considering the equal-sized samples. The estimated IRR from the first model, included in Table 10, indicates that a 1°C increase in the daily maximum temperature elevates the daily average HST count 22.2%, while the level of a working day is maintained at a fixed level. Furthermore, the mean number of HST on a working day decreases by (1–0.865)×100% = 13.5%, compared to that of a weekend or public holiday for constant daily maximum temperature, which highlighted a lower likelihood of heatstroke occurrence during a working day for people younger than 60 years. Younger people are commonly expected to work for the whole day on working days. In some cases, young people need to spend working days outside for construction, transportation, cleaning or other work for which working days are presumed to be more hazardous for young people. The statistics of the Fukuoka City population based on their occupation demonstrate that only 13.5% of the working people of Fukuoka City are engaged in outdoor work, which is quite low as compared to the percentage of indoor workers (75.9%) of the same [33]. Such a salient difference in the proportion of indoor and outdoor workers in the city may have contributed to the results obtained from the models, which seems to be ambivalent. To distinguish the people who were mainly contributing to the association between a working day and heatstroke occurrence, an additional analysis was conducted to divide this younger age group (<60) into two subgroups: (1) patients aged 20 or below and (2) patients aged between 20 and 60. The impact of working days on heatstroke occurrence was not statistically significant for the models used on patients aged between 20 and 60 years. The best-fitted models among those conducted with all possible combinations of main covariates, modified weather-related factors, and working day to reveal a statistically significant impact of working day are also incorporated in Table 10.

Models with 20 years or less aged HST also indicated a negative association between the mean number of HST per day and the number of working days. School, college, or younger university students usually constitute this age group. Students need to participate in various types of intense outdoor sports activities during weekends and holidays, which were responsible for making these days riskier for young people in Fukuoka City. In summary, it can be stated that the most vulnerable group of Fukuoka City, people aged 70 years or older, were not influenced by the day of the week. Therefore, both the working days and holidays were equally hazardous for them.

## 5. Discussion

Heat-related hospitalisation and mortality have become a social issue of crucial importance throughout the world and have attracted much interest in this field. Therefore, heat-related illnesses have become an imperative research topic in recent years. The current study first attempted to scrutinise the relationship between daily temperature and WBGT measures with the number of emergency hospital admissions to Fukuoka City in Japan due to heat-related illnesses. The analyses confirmed a highly significant and positive association of the weather conditions with the daily number of HST, which was consistent with findings from previous studies. For example, a significant temperature-morbidity relationship was found by Ng et al. [8], Ye et al. [36], and Sun et al. [52] through the analyses of ambulance dispatches. Furthermore, Onozuka and Hagihara [13] and Basu [35] identified a positive association between elevated temperature and increased risk of mortality. In this study, the most substantial association with heatstroke occurrence was found with daily maximum temperature and WBGT estimates, rather than the daily means. Daily maximum temperature and WBGT measures were also the focus of the analysis of Akatsuka et al. [53] in different regions of Yamanashi Prefecture in Japan. This study found a positive association between heat disorder incidence rates and daily maximum weather conditions, which contradicts findings from the current study. Heatstroke, which is a severe heat-related illness, occurs due to the maladjustment of the thermoregulation system of an individual for prolonged exposure to high temperature [10]. Hence, the highest temperature of a day is the most likely to disturb the natural thermoregulatory system of the body, which leads to heatstroke occurrence. In the case of WBGT, a standard index for the prevention of heatstroke [54, 55], the pooled influence of a day’s optimum measures of temperature, humidity, wind speed, and solar radiation is reflected by the highest WBGT estimate of a day. The functions of the body’s heat consumption can be potentially disturbed by high temperature and humidity along with strong sun exposure, which was also confirmed by Miyake [56]. This characteristic induced a strong association between daily maximum WBGT estimates and heatstroke occurrence.

Some studies [38–40, 57] of adaptive thermal comfort prompted the researchers to analyse some modified weather-related factors along with daily weather measurements to account for the impact of seasonality on heatstroke occurrence concentrating on the body’s thermal sensation. Two of these modified factors were found to be positively and significantly associated with heatstroke occurrence. These variables are the difference between the target day and the previous day’s average temperature and the difference in the previous nine days’ time-weighted temperature from the target day’s mean temperature. In both cases, the differences were obtained from the average temperature of the target day so that the problem of multicollinearity could be reduced. In contrast, Kotani et al. [22] detected an elevated risk of ambulance dispatches during high temperatures for only the current day and previous day. However, the resulting statistically significant and positive relationship of the two modified weather-related variables from this study, in addition to the daily number of heatstroke patients, revealed how heat acclimation of the human body evolves during consecutive days of the warm season. Heat acclimation refers to the ability of the human body to adapt to heat stress through thermoregulation. A composite interplay between physiological and behavioural factors is responsible for thermoregulation of the human body. Impairment of these factors reduces the body’s thermal tolerance, which puts the thermal adaptation capacity at risk [58, 59]. The higher value of the current day’s average temperature compared to that of the previous day deteriorates the thermal adaptation capacity of the human body, which in turn leads to an increase in the number of heatstroke victims. A similar situation persists for the difference between the average temperature of the current day and the time-weighted temperature of the previous nine days. The higher the difference, the lower the adaptation capacity of a person to the target day’s temperature. Consequently, a salient influence of this temperature difference on heatstroke occurrence was observed.

The breakdown of the heatstroke patients according to a 10-year age structure motivated the classification of the total number of patients into four groups and the repetition of similar analyses in these classified groups identified those who are aged 70 years or older as being the most vulnerable. In Switzerland, Raggettli et al. [28] identified people older than 74 years as those at highest risk of mortality due to rising temperatures. With an increase in age, physiological function begins to decline, which also disrupts the thermoregulation system of the human body. Less sweating and weak cutaneous vascular responses are also responsible for poor thermoregulation in the elderly. In addition, older people are also found to experience greater thermal strain than younger people during heat exposure, which makes elderly people more susceptible [20, 60–63].

The analysis of the characteristics of heatstroke sufferers revealed some important factors that are required to be cautiously considered. The most important of these is the location of the heatstroke occurrence. Different places were determined to have varying degrees of heatstroke risk among people from various age groups. For example, residential areas were depicted to be the riskiest place for the vulnerable group, while younger people were found to face heatstroke mostly in educational institutions. Outside areas and workplaces were places where most of the working people experienced heatstroke. These results successfully project the health hazards in various places. Vulnerable people aged 70 years or above commonly spend most of their time in their houses, which resulted in the highest risk in residential areas for this group. Meanwhile, a survey on consumption trends conducted by the Cabinet Ministry in 2014 reported that the household ownership of air-conditioners (AC) for space cooling was 94.2% in Fukuoka Prefecture. Considering this value, the high proportion of residence as the place of heat stroke occurrence among elderly people shown in Fig 7 implies that they refrained from using AC despite the hot indoor conditions, which also concurs with the findings of *Kayaba et al*. [64] (written in Japanese). This study revealed the reluctance of Japanese elderly people to use AC during sleep. The reasons for reluctance of AC usage among the senior citizens of Japan may include the age-related decline in sensitivity to the heat, the wrong perception regarding the health impact of AC-use, the saving of electric bills, etc. On the contrary, the student community, aged 20 years or below, usually attend day-long classes and also engage in various types of indoor and outdoor sporting events in educational institutions. Outdoor sporting events ensure students’ exposure to higher temperatures during hot summer days, which may substantially affect their thermoregulatory system. Workplaces and outside areas, including outdoor workplaces, are the most frequently used places for people aged between 20 and 60 years which are the working group. People from this age group may also spend holidays or vacations in the sea, shores, mountains, forests, parks, gardens, and other open spaces; which are categorised as outside areas. Prolonged exposure to heat during the warm season makes workplaces and outside places the most hazardous for the working people.

The current study then scrutinised the impact of the corresponding day type, in other words, whether it is a working day or not; on heatstroke occurrence. This variable did not show any significant difference in the mean number of heatstroke patients predicted from the models conducted with patients of all ages and elderly patients. Even in patients aged between 20 and 60 years, no significant association was observed between the type of day and heatstroke occurrence. The significant and negative influence on the heatstroke occurrence of the type of day was observed among the 20-or-lower-aged people, whereas Kotani et al. [22] also observed higher mean numbers of ambulance dispatches for all ages during weekend or holidays compared to working days. The finding from the current study indicates that the student community of Fukuoka City is responsible for such an emerging and significant difference in heatstroke occurrence, and these students are determined to experience a lower probability of ensuing heatstroke during working days. However, working days are presumed to be more hazardous for heatstroke than weekends or public holidays because working people are expected to endure prolonged heat exposure during these days. Working days have an adverse impact on outdoor workers, such as those involved in agriculture, construction, mining, and manufacturing industries; along with armed forced personnel and firefighters [65]. The proportion of outdoor workers is relatively low in Fukuoka City, while the service sector is dominant among all occupations, which is the rationale for the controversial association between working day and heatstroke occurrence. Moreover, students need to attend various types of sporting events, including outdoor events during weekends or on public holidays, which necessitates prolonged heat exposure for them. For this reason, weekends or public holidays emerged as being more hazardous concerning heatstroke for people aged 20 years or less as compared to working days.

This study has some limitations that must be acknowledged. Firstly, the findings of this study cannot be generalised to the entire population of Japan as it was a single-city study. Moreover, the vulnerability of the population can be assessed more precisely if the further decomposition of the heatstroke patients accounts for gender, level of heatstroke or other classification criteria along with age structure. However, such a breakdown would result in a very small sample size, making the reliability of the results obtained from the models questionable.

## 6. Conclusion

Heat-related health issues have increased in many geographic regions because of an upward shift in global temperature over the last three decades, which has made many more countries worldwide highly prone to confront such heat-related illnesses. Heatstroke patients in Fukuoka City, Japan were analysed in this study to investigate potential underlying factors for increased risk of heatstroke. It also aimed to detect the group who was most vulnerable to heatstroke by comparing the susceptibility among heatstroke patients of different ages in this city. Associations were found between the daily maximum weather conditions, such as temperature and WBGT, and an elevated risk of heatstroke. Furthermore, a salient increase in the average temperature from both the previous day’s mean temperature and the consecutive past nine days’ time-weighted temperature also was a significant risk factors of heatstroke. The findings of this study determined the elderly of Fukuoka City in Japan, aged 70 years or older, to be the most vulnerable group for heatstroke. An increased risk of heatstroke was observed for not only the daily weather conditions (temperature and WBGT), but also the substantial temperature rise in the previous nine days for vulnerable groups in the study area. The highest heat-health risk in the residential area was also found for the vulnerable group. Higher hazard on weekends or holidays was revealed for young people aged 20 or below, whereas vulnerable people had equal risk on both working days and weekends or holidays through this study.

The results of this study can provide valuable insights into the health policy implications of Fukuoka City to reduce heat-related health issues. Thus, the local government of this city can design appropriate preventive measures against heatstroke based on the results of the analyses. For instance, some educational campaigns should be designed to enhance people’s awareness and the importance of checking weather forecasts and to take precautions against heat-related illness when a substantial increase in temperature from the past several days is predicted. Some special interventions, such as educational campaigns or advertisements should be introduced focusing on the most vulnerable population of Fukuoka City. The existing heat health warning system, which focuses on high daily temperatures or heat-index like WBGT, needs to be updated to address the risk of heatstroke for a forecast with a salient rise in temperature from previous days. In such situations, a special alert should be sent en masse to people from the government through channels, such as emails, government emergency radio systems, television broadcast, and text messages, which can be implemented on a prefectural basis. These interventions should safeguard elderly people by making them understand the importance of checking weather forecasts and taking adequate preventative measures against heatstroke, such as avoiding going outside, use of air conditioning (AC) in one’s home, and increasing fluid intake. Special intensive care for these vulnerable people should be prepared in advance if extensively high temperature and/or a significant temperature increase from past days is predicted. This group should be encouraged to use ACs in their residences through varying interventions, and some incentives can be designed to enhance AC usage among them. Furthermore, the housing conditions of these people need to be investigated thoroughly in order to reduce the risk of heatstroke occurrence while staying in their residences. The equal seriousness of working days and weekends or holidays for the elderly should also be highlighted through these programmes. The outdoor sporting events of students should be moved to the cooler autumn or spring seasons and can also be replaced by indoor sporting events to avoid the risk of heatstroke. Even though the younger generation is relatively strong and more resistant to heatstroke, large sporting events during hot days may lead to a surge in patients, which should be conceived by health service providers along with policymakers.

The findings of this study are not limited to the areas of this city and Japan, a country experiencing a super-aging society. Many populous countries, due to decreasing levels of fertility and mortality, will also encounter a significant shift in their demographic structure, moving from a youthful generation towards an older one. Such a situation ensures the applicability of the methodologies of this study in various areas throughout the world, which is a potential future scope of this work. Moreover, it will assist healthcare providers in the early allocation of sufficient resources, such as proper treatments for heatstroke sufferers and some special intensive care for people at higher risk. Further studies are required to explore the seasonal influence, accounting for the division of the summer season, such as early, mid, and late summer; on heatstroke occurrence in a more specific manner. Furthermore, the consideration of gender differentials regarding general health state, body mass index, certain baseline diseases, etc. for the vulnerable group of heatstroke may be of profound interest for future studies. Risk assessment for different types of buildings, such as residential, educational, public, and hospital buildings along with the maintenance of heating, ventilation, and AC systems [66] also can be analysed in future studies. This can provide important information regarding how these buildings can be improved to safeguard against heatstroke. In light of the current pandemic, additional studies are also required to investigate whether wearing a mask to prevent the spread of human droplets [67, 68], as a preventive measure against COVID-19 infection, increases the risk of heat-related illness on hot summer days.

## Supporting information

S1 Data Data with daily no. of patients and weather conditions. (CSV)
